# Transcriptome-Wide Identification of Salt-Responsive Members of the *WRKY* Gene Family in *Gossypium aridum*

**DOI:** 10.1371/journal.pone.0126148

**Published:** 2015-05-07

**Authors:** Xinqi Fan, Qi Guo, Peng Xu, YuanYong Gong, Hongmei Shu, Yang Yang, Wanchao Ni, Xianggui Zhang, Xinlian Shen

**Affiliations:** 1 State Key Laboratory of Crop Genetics & Germplasm Enhancement, Nanjing Agricultural University, Nanjing, China; 2 Key Laboratory of Cotton and Rapeseed (Nanjing), Ministry of Agriculture, P. R. China, The Institute of Industrial Crops, Jiangsu Academy of Agricultural Sciences, Nanjing, China; Fujian Agriculture and Forestry University, CHINA

## Abstract

WRKY transcription factors are plant-specific, zinc finger-type transcription factors. The WRKY superfamily is involved in abiotic stress responses in many crops including cotton, a major fiber crop that is widely cultivated and consumed throughout the world. Salinity is an important abiotic stress that results in considerable yield losses. In this study, we identified 109 WRKY genes (*GarWRKYs*) in a salt-tolerant wild cotton species *Gossypium aridum* from transcriptome sequencing data to elucidate the roles of these factors in cotton salt tolerance. According to their structural features, the predicted members were divided into three groups (Groups I–III), as previously described for *Arabidopsis*. Furthermore, 28 salt-responsive *GarWRKY* genes were identified from digital gene expression data and subjected to real-time quantitative RT-PCR analysis. The expression patterns of most *GarWRKY* genes revealed by this analysis are in good agreement with those revealed by RNA-Seq analysis. RT-PCR analysis revealed that 27 *GarWRKY* genes were expressed in roots and one was exclusively expressed in roots. Analysis of gene orthology and motif compositions indicated that WRKY members from *Arabidopsis*, rice and soybean generally shared the similar motifs within the same subgroup, suggesting they have the similar function. Overexpression-*GarWRKY17* and –*GarWRKY104* in *Arabidopsis* revealed that they could positively regulate salt tolerance of transgenic *Arabidopsis* during different development stages. The comprehensive data generated in this study provide a platform for elucidating the functions of WRKY transcription factors in salt tolerance of *G*. *aridum*. In addition, *GarWRKYs* related to salt tolerance identified in this study will be potential candidates for genetic improvement of cultivated cotton salt stress tolerance.

## Introduction

Plants have developed a series of complex, effective systems to protect themselves from a variety of adverse environmental conditions during growth over the long course of evolution [1]. Studies have shown that these coping mechanisms mainly function via transcriptional activation or inhibition of transcription-related genes. Transcription factors play an important role in this process by activating or inhibiting the expression of target genes alone or via interactions with other proteins [2–4].

WRKY transcription factors are recently identified plant-specific transcription factors comprising one or two WRKY domains and a zinc-finger motif. The name WRKY is derived from the highly conserved WRKY domain, which consists of 60 amino acids. The WRKY domain comprises the highly conserved WRKYGQK sequence followed by a novel zinc-finger motif (C_2_H_2_orC_2_HC) located in the N-terminus [5]. Based on the number of WRKY domains and the pattern of zinc-finger motifs, WRKY proteins can be classified into three main groups and eight subgroups [5]. Group I WRKYs typically have two WRKY domains containing a C_2_H_2_ zinc-finger motif. The C-terminal WRKY domain has DNA binding activity. However, the N-terminal WRKY domain cannot bind to DNA alone, but it can assist the C-terminal WRKY domain in its binding to DNA, increasing its DNA binding affinity and specificity. In addition, the zinc-finger motif followed by the N-terminal WRKY domain may provide a protein–protein interaction interface; the zinc-finger motif’s structure is C_2_H_2_ (C-X_4–5_-C-X_22–23_-H-X-H, X is any amino acid). Group II WRKYs have a single WRKY domain with a C_2_H_2_ zinc-finger motif, which is the same as the C-terminal domain of Group I. Based on the phylogeny of the WRKY domains, group II WRKYs can be further divided into five subgroups: IIa (C-X_5_-CX_23_-HXH), IIb (C-X_5_-CX_23_-HXH), IIc (C-X_4_-CX_23_-HXH), IId (C-X_5_-CX_23_-HXH) and IIe (C-X_5_-CX_23_-HXH). Group III WRKYs also have a single WRKY domain, but their zinc-finger motif structure is C_2_HC (C-X_7_-C-X_23_-H-X-C). Based on the zinc finger structure, Group III can be further divided into two subgroups: IIIa and IIIb. The structure of subgroup IIIa zinc fingers is C-X_7_-C-X_23_-HXC, while that of subgroup IIIb is C-X_7_C-Xn-HXC (n ≥ 24) [6]. WRKYs specifically bind to the W-box, i.e., (T) (T) TGAC (C/T), in the promoter regions of target genes to regulate their transcription levels, enabling them to perform their biological functions [5].

Since the first report of *SPF1* WRKY transcription factors from sweet potato in 1994 [7], numerous WRKY transcription factors have been experimentally identified from many other plant species including *Arabidopsis*, rice, tobacco, potato, cotton, barley, wheat, chamomile, soybean, cacao, grape, tomato, cucumber and so on [8–20]. Except for plant development, WRKY family genes also play important roles in plant biotic and abiotic stress responses including pathogen-induced defense programs and responses to drought, salt, stress et al.[20–22]. One of the most important functions of WRKYs appears to be regulation of the salt stress response. For example, *AtWRKY25* and *AtWRKY33* play a role in the salt stress response and improve salt tolerance in *Arabidopsis* [23]. *OsWRKY11* and *OsWRKY45* play a regulatory role in salt tolerance in transgenic rice [24,25]. *GmWRKY54*-overexpressing plants are more salt and drought tolerant than the wild type, and *GmWRKY13* overexpression results in increased sensitivity to salt and mannitol stress [26]. Finally, overexpression of *TaWRKY10* enhances drought and salt tolerance in transgenic tobacco plants [27].

In recent years, some WRKY transcription factors have also been reported in cotton. Guo et al. isolated *GhWRKY3* from upland cotton (*G*. *hirsutum*), which is upregulated by the application of various phytohormones but not by cytokinin, auxin analog, drought, NaCl or cold [18]. Yu et al. identified *GhWRKY15* from *G*. *hirsutum*, which is involved in disease resistance and plant development [28]. Shi et al. isolated a putative IId WRKY gene, *GhWRKY39*, from *G*. *hirsutum* and showed that it is activated by pathogens and salt stress. *GhWRKY39*-overexpressing *Arabidopsis* plants display enhanced tolerance to salt [29]. Zhou et al. identified 26 genes encoding putative WRKY proteins in *G*. *hirsutum*; five *GhWRKY* genes are upregulated in roots treated with NaCl [30]. With the release of the *G*. *raimondii* genome sequence, two comprehensive studies of *G*. *raimondii* WRKY family members were conducted, revealing 120 and 116 *GrWRKY* genes and 109 WRKY genes in *G*. *arboreum* [31–33], respectively; the expression of these genes under abiotic stress was analyzed in *G*. *hirsutum* [31,32]. All of these results suggest that WRKY genes are involved in the response to salinity stress in cotton.

High salinity is a major abiotic stress in cotton production worldwide. However, as described in detail previously [34], modern cotton cultivars, which are often grown under unstressed conditions, are the result of intensive selection to produce large amounts of specific types of fiber. Selection has unintentionally narrowed the genetic variability for salt tolerance. *G*. *aridum* is a D-genome diploid species from the Pacific coastal states of Mexico that shows remarkably tolerance to salt stress. Understanding how *G*. *aridum* responds to and develops tolerance to salt stress is the first step towards improving the adaptation of cotton to salt stress. In a previous study, we performed deep sequencing analysis of the *G*. *aridum* transcriptome in response to salt stress. A total of 281 unigenes were annotated as WRKY family members. Differentially expressed genes encoding transcription factors belonging to the WRKY family were predominant during different stages of salt stress [34]. These results suggest that WRKY family members are important regulators of the response to salt stress in *G*. *aridum*. In the current study, we focused on whole transcriptome-wide identification of salt-responsive members of the WRKY family in *G*. *aridum*. The results of this study increase our understanding of the *GarWRKY* gene family, which will facilitate the genetic improvement of salt stress tolerance in cotton.

## Materials and Methods

### Plant materials and treatment conditions

Seeds from the wild *Gossypium* species *G*. *aridum* were kindly supplied by the National Wild Cotton Plantation in Hainan Island, China. The same treatment procedure was used as described in Xu et al. [34]. *G*. *aridum* plants were treated with 200 mM NaCl for 0, 1, 3, 6, 12, 24 and 72 h. Root and leaf tissues were collected at every stage of stress treatment. All tissues were immediately frozen in liquid nitrogen and stored at −70°C.

### *GarWRKY* gene identification and chromosomal location

The annotated genome sequences of *G*. *raimondii* were downloaded from http://www.phytozome.net/cn/. The WRKY domain (PF03106) downloaded from PFAM protein family database was used as a query for identifying WRKY transcription factors by HMMER software version 3.0[35]. The annotated *G*. *raimondii* WRKY genes were used to establish a local nucleic acid database. Based on de novo transcriptome sequencing data with a pooled RNA sample from roots and leaves of 12 h salt-stressed and unstressed plants [34], more than 90,000 transcripts were identified and used to BLAST the *G*. *raimondii* WRKY gene database using a local BLAST program with E-value < 10^−10^. Redundant sequences were removed. The GarWRKY protein sequences were further analyzed to confirm the presence of WRKY domains using the InterPro program (protein sequence analysis & classification, http://www.ebi.ac.uk/interpro/) [36].

To determine the location of *GarWRKY* genes on chromosomes, the *GarWRKY* sequences were further used as query sequences for a BLASTN search against *G*. *raimondii* whole-genome scaffold data (http://www.phytozome.net). Finally, the locations of all 109 *GarWRKY*s in the *G*. *raimondii* genome were detected. Mapping of *GarWRKY* genes was performed using MapInspect (http://www.plantbreeding.wur.nl/UK/software_mapinspect.html). To better identify and classify the WRKY transcription factors (TFs) in *Gossypium*, a uniform nomenclature was assigned to the WRKY genes in *G*. *aridum* as in *G*. *raimondii* [31].

### *GarWRKY* gene classification and phylogenetic analysis

To classify *GarWRKY* genes into different groups and subgroups, a total of 14 *AtWRKY* genes representing three main groups and seven subgroups were selected, including Group I *(AtWRKY7* and *AtWRKY28*), Group IIa (*AtWRKY25* and *AtWRKY27*), Group IIb (*AtWRKY28* and *AtWRKY36*), Group IIc (*AtWRKY40* and *AtWRKY41*), Group IId (*AtWRKY42* and *AtWRKY49*), Group IIe (*AtWRKY58* and *AtWRKY60*) and Group III (*AtWRKY69* and *AtWRKY70*). Complete WRKY domains from all GarWRKY proteins and select AtWRKY proteins were used to align WRKY domains with ClustalW [37]. The consequential alignment was used to create a phylogenetic tree in MEGA v4.0 using the neighbor-joining (NJ) method with 1,000 bootstrap replications [38].

To assess structural divergence of WRKY genes in different species, the Multiple Expectation Maximization for Motif Elicitation program (MEME, ttp://meme.nbcr.net/meme/tools/meme) was used to identify conserved motifs in the encoded proteins. Parameters employed in the analysis were: minimum motif width, 6; maximum motif width, 50; and maximum number of motifs, 10.

### Isolation of salt-induced WRKY family genes

In a previous study, digital gene expression (DGE) analysis of *G*. *aridum* under salt stress revealed that the expression levels of 28 of 109 WRKY genes were significantly altered under salt stress. Using gene-specific primers (S2 Table) designed based on corresponding homologous gene in the *G*. *raimondii* genome, WRKY genes were cloned by PCR and their transcripts were amplified from *G*. *aridum* roots treated with 200 mM NaCl for 12 hours. PCR was performed using EasyPfu DNA Polymerase (TransGen Biotech, China). The PCR products were cloned into the PTG19-T vector (TransGen Biotech, China) and sequencing was performed by Invitrogen (Shanghai, China). The subcellular localizations of proteins were predicted by CELLO v.2.5 (subcellular localization predictor, http://cello.life.nctu.edu.tw) [39].

### Expression analysis of salt-induced *GarWRKY* members *in G*. *aridum*

#### RNA isolation

Total RNA from *G*. *aridum* leaves and roots was extracted using an improved CTAB method [40]. For RT-PCR, first-strand cDNA was synthesized with a BU-SuperScript RT Kit (Biouniquer, China) according to the manufacture’s protocol. For quantitative real-time PCR, first-strand cDNA was synthesized with PrimeScript RT Master Mix (Perfect Real Time) from TaKaRa (Dalian, China).

#### Semi-quantitative RT-PCR

Semi-quantitative RT-PCR was performed to detect the differential expression of *GarWRKY* genes in different tissues including root, stem, leaf, petal, stigma, stamen and calyx tissue under normal conditions. The RT-PCR condition were as follow: preheating at 94°C for 3 min, followed by 36 cycles of 95°C for 45 s, 60°C for 45 s and 72°C for 1 min, with a final extension at 72°C for 10 min to complete the reaction. The PCR products were separated on a 1.2% agarose gel and quantified using an Imaging System (Bioshine GelX 1520, China). The specific primers used for RT-PCR are listed in S3 Table. The cotton actin gene was used as an internal reference gene (S3 Table).

#### Quantitative real-time PCR

The qRT-PCR was performed on an ABI PRISM 7500 Real-Time PCR System. The qRT-PCR reaction system (20 μl) contained 10 μl 2×SYBR Premix Ex Taq, 1 μl cDNA temple, 0.4 μl each of forward and reverse primer (10 μΜ) and 0.4 μl ROX Reference Dye II (50×). The thermal cycling conditions were as follow: pre-denaturation at 95°C for 30 s, followed by 40 cycles at 95°C for 5 s and 60°C for 34 s. After the cycle was complete, melting curves analysis was performed at 60–95°C to verify the specificity of the amplicon for each primer pair. Quantitative real-time PCR was carried out with three biological replicates. The cotton actin gene was used as an internal reference gene. The specific primers used for qRT-PCR are listed in S3 Table. The relative gene expression values were analyzed by the 2^-ΔΔt^ method.

#### Generation of *GarWRKY17* and *GarWRKY104* transgenic *Arabidopsis* plants

The cDNA sequence containing the full-length coding sequence of *GarWRKY17* and *GarWRKY104* were cloned into pCAMBIA2301 expression vector separately under the control of CaMV35S promoter between the *Xba* I and *Kpn* I sites. The recombinant plasmid pCAMBIA2301-*GarWRKY17* and pCAMBIA2301-*GarWRKY104* were transformed into *Agrobacterium* EH105 with electroporation method (V = 2.4kv). The *Arabidopsis* plants were transformed by the floral dip method [41]. Their T_2_ transgenic lines were obtained after screening of Kanamycin resistance, PCR and RT-PCR analysis.

#### Analysis of salt tolerance in transgenic *Arabidopsis* plants

To evaluate salt tolerance of *GarWRKY17* and *GarWRKY104* transgenic *Arabidopsis* plants during seed germination stage, 50 sterilized seeds of wide type (WT) and T_2_ generation transgenic lines (3 lines for *GarWRKY17* and 2 lines for *GarWRKY104*) were sowed in MS medium with and without 150 mM NaCl. The experiment was conducted with three biological replicates. Germination rate was calculated after 10 days.

To obtain further evidence that whether the overexpression of *GarWRKY17* and *GarWRKY104* could confer resistance to salt stress during vegetative growth, sterilized seeds of WT and T_2_ transgenic *Arabidopsis* were sowed in soil and grew for 20 days. Plants were growing in a pot supplemented with 150ml water or 150mM NaCl. Phenotype symptoms were observed after 4 weeks.

To determine the activities of antioxidant enzymes, three-week-old WT seedlings and T_2_ seedlings of 2 *GarWRKY17*–overexpressing (*GarWRKY17*-10 and *GarWRKY17*-25) were exposed to 150 mM NaCl for 24h. Leaves were collected from 10 plants of wild type and two transgenic lines. The activities of superoxide dismutase (SOD) and peroxidase (POD) was assayed using the procedure described by Liu et al. [42]. The tests were performed in triplicate.

## Results

### Identification of WRKY genes in *G*. *aridum* and their chromosomal locations

**Fig 1:**
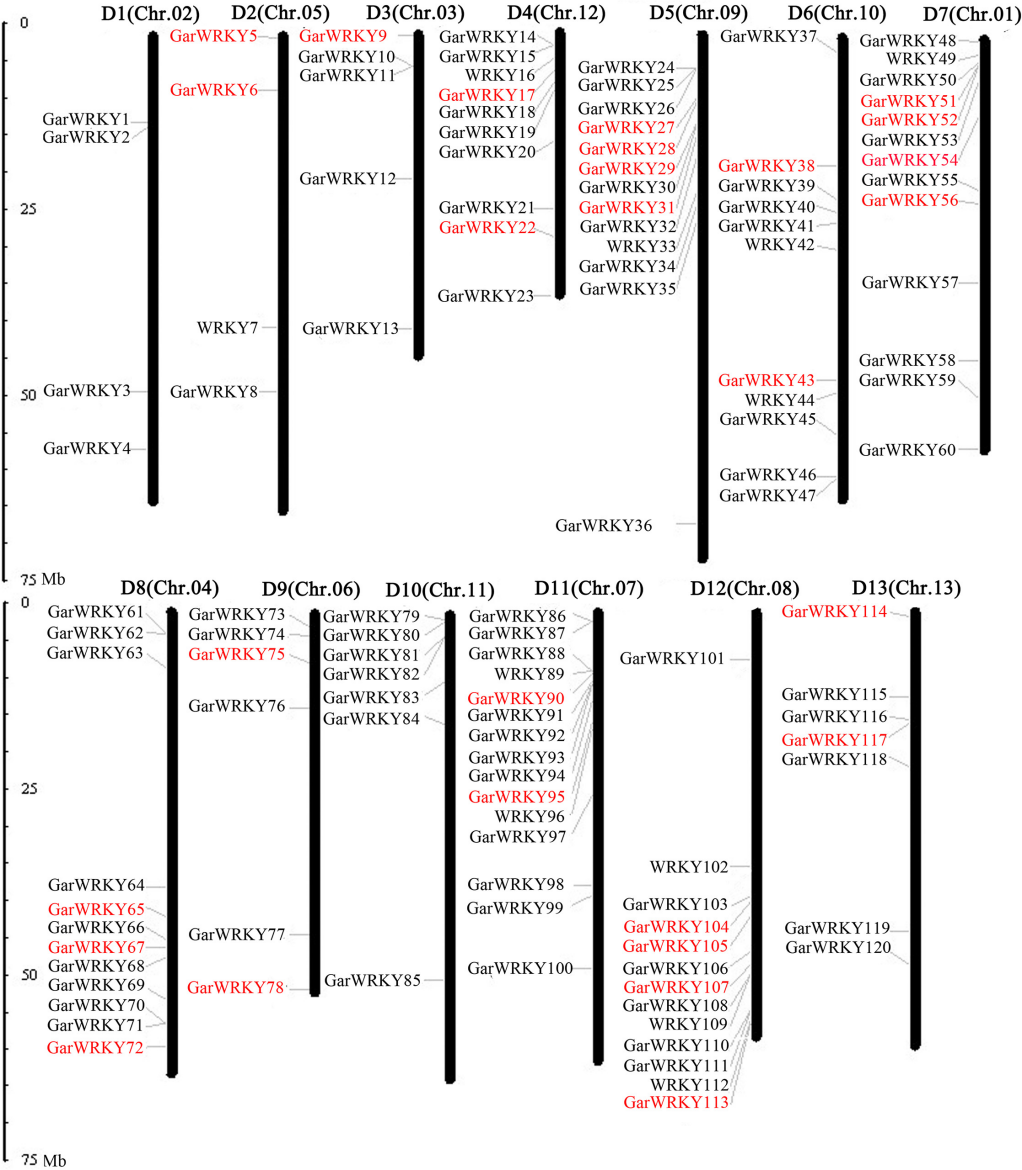
Chromosomal locations of *GarWRKY* genes in the *G*. *raimondii* genome. The candidate WRKY genes in *G*. *aridum* were designated *GarWRKY1* to *GarWRKY120* based on their orders on chromosomes. Eleven WRKY genes in *G*. *aridum* (*GarWRKY7*, 16, 33, 42, 44, 49, 89, 96, 102, 109 and 112) not identified in the current study were replaced by corresponding WRKY genes in the *G*. *raimondii* genome based on a report by Cai et al. (2014). Salt-responsive *GarWRKYs* are indicated in red. The scale is in megabases (Mb).

In a previous study, we identified more than 90,000 transcripts from de novo transcriptome sequencing of *G*. *aridum* under salt stress [34]. To perform genome-wide analysis of WRKY genes in *G*. *aridum*, all of these transcripts were used to BLAST the *G*. *raimondii* WRKY gene database using a local BLAST program. A total of 109 genes in *G*. *aridum* were ultimately identified as possible members of the WRKY superfamily and named according to their position from the top to the bottom of cotton chromosomes 1–13 (Fig 1). To facilitate communication, a uniform nomenclature for the 109 *GarWRKY* genes identified in this work was adopted based on that used in *G*. *raimondii* by Cai et al. [31]. The authors identified 120 candidate WRKY genes in the *G*. *raimondii* genome. By comparison, 11 WRKY genes (*WRKY7*, 16, 33, 42, 44, 49, 89, 96, 102, 109 and 112) were not identified in the current study.

*GarWRKY* genes are distributed on all 13 *G*. *aridum* chromosomes. Of these, chromosome 7 has the highest number of *GarWRKY* genes (13) and chromosome 5 has the lowest (3). The distribution patterns of these genes on individual chromosomes reveal some regions with numerous *GarWRKY* gene clusters. For example, *GarWRKY* genes located on chromosomes 4, 8, 9 and 11 appear to be concentrated on the lower or upper ends of the arms of chromosomes, respectively (Fig 1).

### Classification and phylogenetic analysis of *GarWRKY* genes

The WRKY domain and the zinc finger motif are the most prominent structural features of WRKYs. The WRKY domain contains the highly conserved heptapeptide stretch WRKYGQK at its N-terminus followed by a zinc-finger motif. A total of 126 WRKY domains were found in the 109 GarWRKY protein sequences.

**Fig 2:**
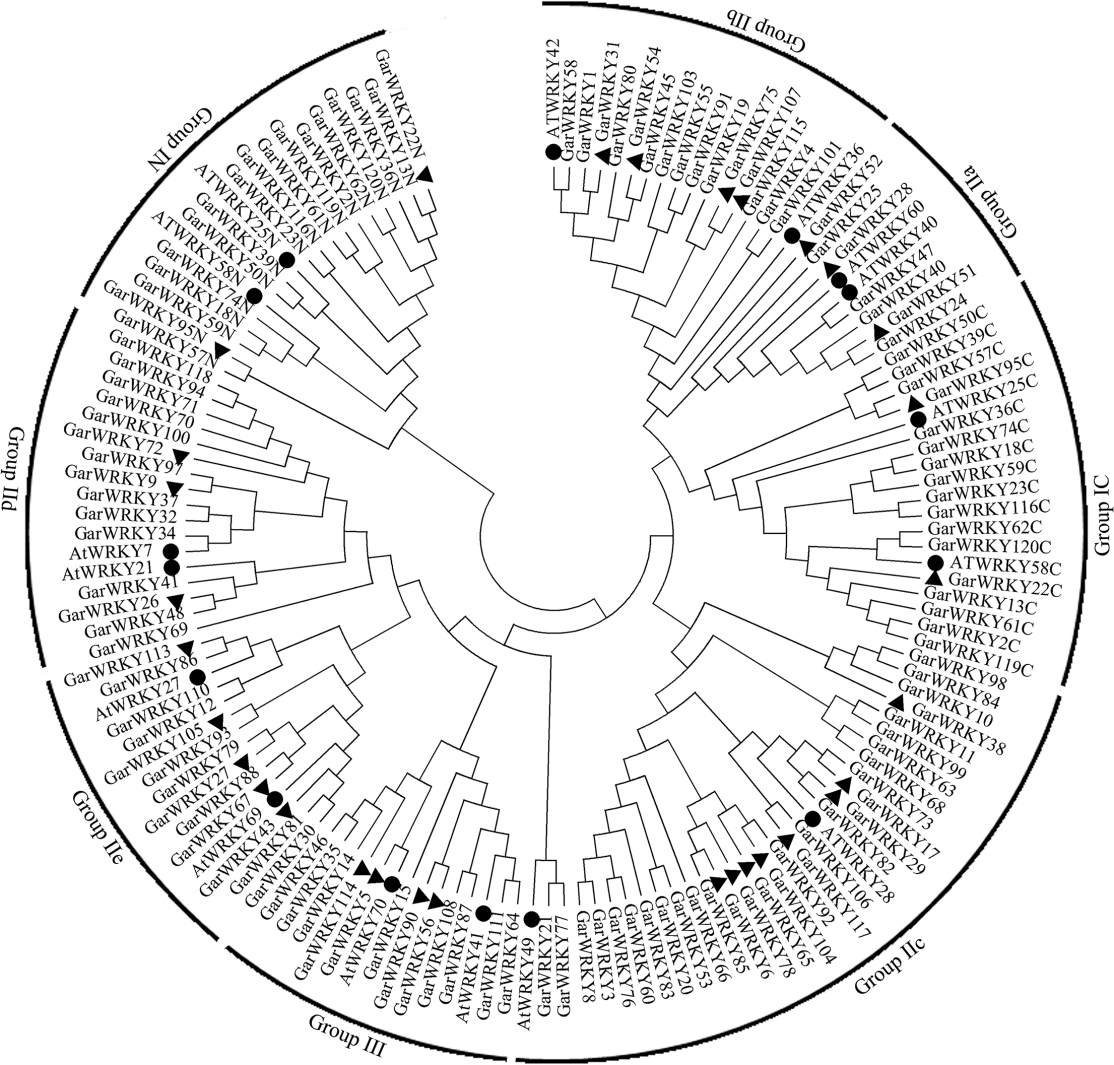
Phylogenetic analysis of WRKY domains in *G*. *aridum*. WRKY protein name with the suffix ‘N’ or ‘C’ indicates the N-terminal WRKY domains or the C-terminal WRKY domains. The black arcs indicate different groups of WRKY domains. ● represent AtWRKY proteins, ► represent salt-responsive GarWRKY proteins.

The amino acid sequences of 126 WRKY domains were aligned using ClustalW with default settings. Based on AtWRKY classification and the WRKY domain features of GarWRKYs, the GarWRKYs were mainly classified into three groups, designated Group I to Group III (Fig 2). Seventeen GarWRKYs were assigned to Group I; these proteins contain two WRKY domains, an N-terminal WD (NTWD) and a C-terminal WD (CTWD). The NTWD zinc-finger type is C-X_4_-C-X_22_-HXH and the CTWD zinc-finger type is C-X_4_-CX_23_-HXH. Eighty GarWRKYs were assigned to Group II, which contain a single WRKY domain. The zinc-finger type is also C_2_H_2_, but its structure is C-X_4–5_-C-X_23_-HXH, which is structurally different from the Group I zinc finger motif. These 80 Group II GarWRKYs were further divided into five subgroups: subgroup IIa (7), IIb (15) IIc (30), IId (15) and IIe (13). Twelve GarWRKYs were assigned to Group III; these proteins contain a single WRKY domain with a C_2_HC zinc-finger motif (C-X_7_-C-X_23_-HXC). Detailed information about the GarWRKYs can be found in S1 Table.

### Cloning and classification of salt-induced WRKY family genes

In a previous study, we performed digital gene expression profile analysis of *G*. *aridum* during different stages of salt stress and identified 28 WRKY ESTs that were differentially expressed in roots or leaves during at least one stage [34]. In the current study, using gene-specific primers designed based on the corresponding homologous genes in the *G*. *raimondii* genome, we performed PCR cloning of WRKY genes and amplified the transcripts from roots of *G*. *aridum* treated with 200 mM NaCl for 12 h. We obtained 28 cDNA sequences of WRKY genes with complete open reading frames (ORFs; GenBank accession numbers KM438453–KM438480; Table 1). The ORF length ranged from 474 bp to 1,848 bp, with an average length of 1,048 bp. The identified *GarWRKY* genes encode proteins ranging from 157 to 615 amino acids (aa) in length with an average of 348 aa. The similarities between *GarWRKY* and *GrWRKY* based on their full-length nucleotide sequences ranged from 93.5% to 99.88%. All 28 salt-responsive GarWRKY proteins were predicted to be located in the nucleus.

**Table 1:** Characterization of 28 salt-responsive *GarWRKY* members.

GarWRKY	Group	ORF (bp)	Polypeptide (aa)	GrWRKY	Similarity (%)	Subcellular localization	GenBank accession number
GarWRKY5	III	921	306	WRKY5	98.05	Nuclear	KM438453
GarWRKY6	IIc	1077	358	WRKY6	99.07	Nuclear	KM438454
GarWRKY9	IId	951	316	WRKY9	98.74	Nuclear	KM438455
GarWRKY17	IIc	957	318	WRKY17	98.03	Nuclear	KM438456
GarWRKY22	I	1530	509	WRKY22	99.22	Nuclear	KM438457
GarWRKY27	IIe	1386	461	WRKY27	99.49	Nuclear	KM438458
GarWRKY28	IIa	933	310	WRKY28	99.41	Nuclear	KM438459
GarWRKY29	IIc	960	319	WRKY29	99.06	Nuclear	KM438460
GarWRKY31	IIb	1698	565	WRKY31	99.04	Nuclear	KM438461
GarWRKY38	IIc	474	157	WRKY38	99.37	Nuclear	KM438462
GarWRKY43	IIe	828	275	WRKY43	98.56	Nuclear	KM438463
GarWRKY51	IIa	876	291	WRKY51	93.5	Nuclear	KM438464
GarWRKY52	IIa	786	261	WRKY52	98.85	Nuclear	KM438465
GarWRKY54	IIb	978	325	WRKY54	98.56	Nuclear	KM438466
GarWRKY56	III	1008	335	WRKY56	99.01	Nuclear	KM438467
GarWRKY65	IIc	867	288	WRKY65	99.88	Nuclear	KM438468
GarWRKY67	IIe	978	325	WRKY67	99.39	Nuclear	KM438469
GarWRKY72	IId	1020	339	WRKY72	97.65	Nuclear	KM438470
GarWRKY75	IIb	1443	480	WRKY75	98.83	Nuclear	KM438471
GarWRKY78	IIc	885	294	WRKY78	98.8	Nuclear	KM438472
GarWRKY90	III	999	332	WRKY90	98.8	Nuclear	KM438473
GarWRKY95	I	1215	404	WRKY95	99.42	Nuclear	KM438474
GarWRKY104	IIc	966	321	WRKY104	98.65	Nuclear	KM438475
GarWRKY105	IIe	717	238	WRKY105	99.02	Nuclear	KM438476
GarWRKY107	IIb	1848	615	WRKY107	98.11	Nuclear	KM438477
GarWRKY113	IIe	1194	397	WRKY113	98.49	Nuclear	KM438478
GarWRKY114	III	876	291	WRKY114	95.97	Nuclear	KM438479
GarWRKY117	IIc	786	261	WRKY117	99.46	Nuclear	KM438480

### Expression patterns of *GarWRKY* genes in various tissues revealed by semi-quantitative RT-PCR analysis

**Fig 3:**
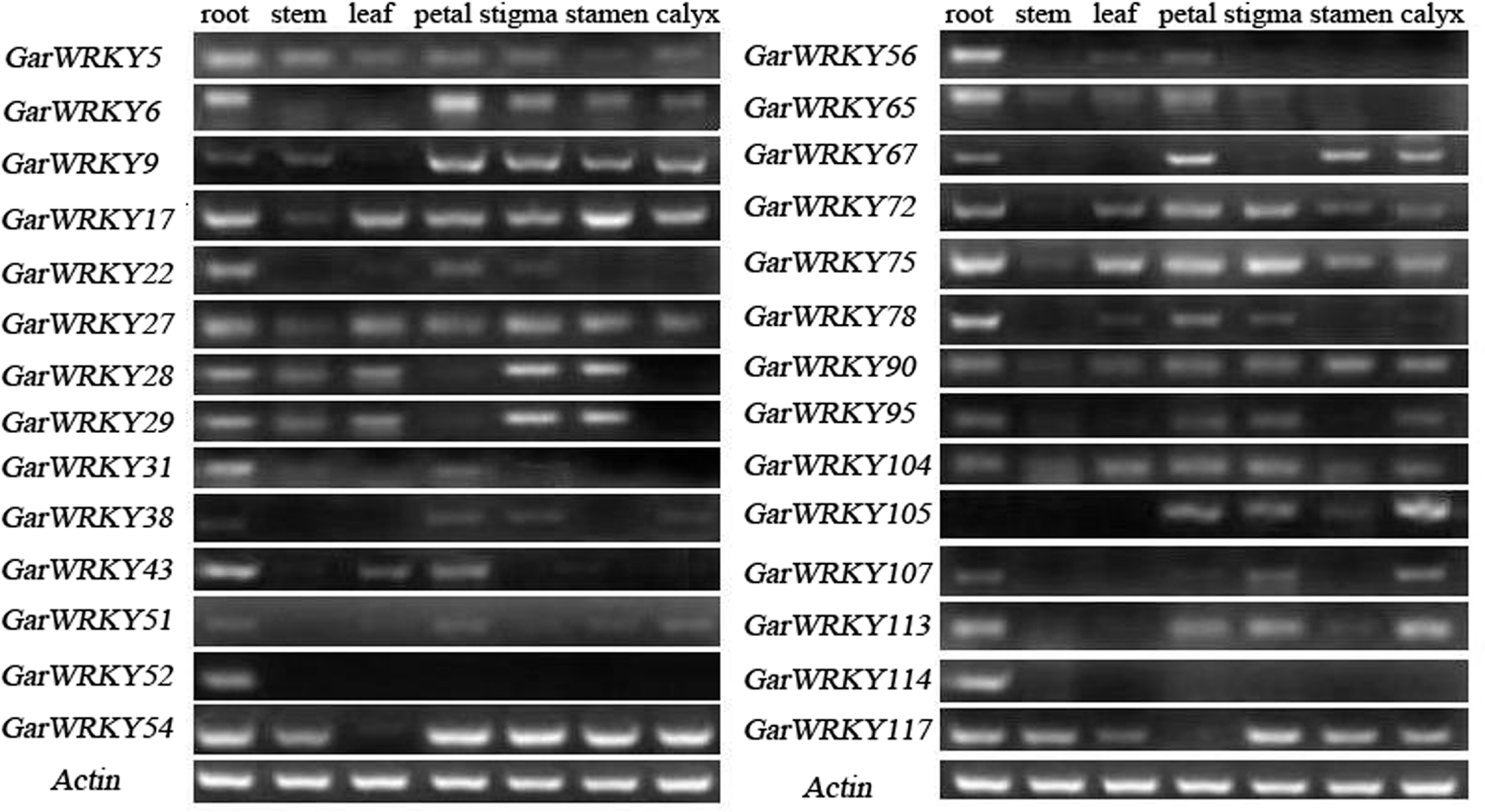
Expression patterns of *GarWRKY* genes in various tissues. Semi-quantitative RT-PCR was conducted under normal growth condition. The cotton actin gene was used as an internal reference.

Using RT-PCR, we examined the expression patterns of *GarWRKY* genes in plants grown under normal growth conditions in seven different tissues including root, stem, leaf, petal, stigma, stamen and calyx tissue. As shown in Fig 3, 27 *GarWRKY* genes were expressed in roots, while 6 genes (*GarWRKY5*, *GarWRKY17*, *GarWRKY27*, *GarWRKY75*, *GarWRKY90* and *GarWRKY104*) were expressed in all tissues (with varying expression levels). Moreover, 21 genes had different tissue-specific expression profiles and *GarWRKY51* had very low transcript abundance in all tissues. Notably, *GarWRKY*52 was exclusively expressed in roots.

### Validation of significant changes in *GarWRKY* expression in response to salt stress using real-time PCR

**Fig 4:**
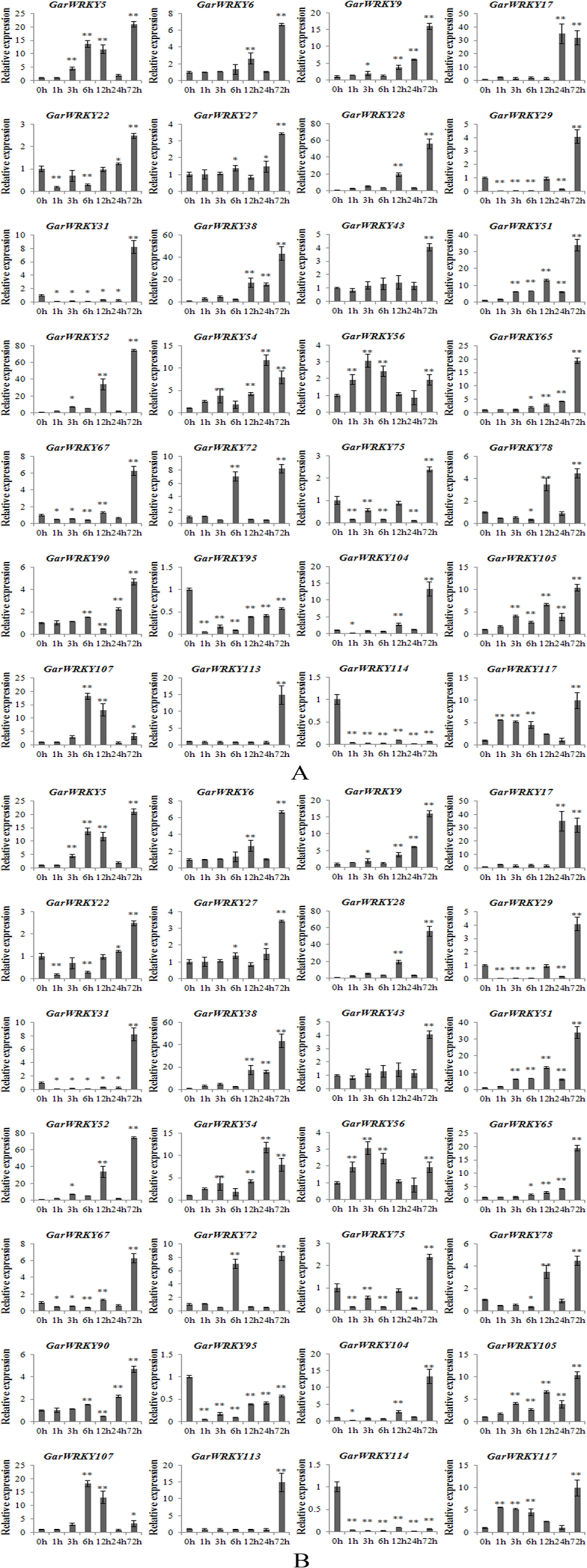
Expression profiles of *GarWRKY* genes under NaCl stress. A: roots; B: leaves. The cotton actin gene was used as an internal reference. The data are mean ± SE of three biological replicates. * and ** indicate statistical significance at the 0.05 and 0.01 probability level, respectively.

Gene expression patterns can provide important clues for determining gene function. To validate the results obtained from RNA-Seq, we performed quantitative RT-PCR analysis (Q-PCR) of the differentially expressed *GarWRKY* genes to confirm their levels of expression in roots and leaves after salt stress treatment. Quantitative expression analysis of these genes was performed using samples obtained from independent experiments carried out under the same conditions used to obtain RNA samples for RNA-Seq analysis. RNA was extracted from roots and leaves at 0, 1, 3, 6, 12, 24 and 72 h post salt stress, while RNA-Seq analysis was performed with fewer time points (0, 3, 12 and 72 h). As shown in Fig 4, the *GarWRKY* genes displayed distinct expression patterns in response to salt stress. Real-time PCR generally produced similar results to those obtained by differential expression profiling by RNA-Seq. However, in contrast to the RNA-Seq results, qRT-PCR revealed reduced expression of *GarWRKY95* and *GarWRKY114* in leaves.

Most *GarWRKY* genes in roots were activated within 1 h following salt treatment, with peak expression during the middle period and a return to basal levels during the latest period (72 h; Fig 4). However, in leaves, the majority of the analyzed WRKY genes were activated later than 6 h after salt treatment, with peak expression during the latest period (72 h). Expression analysis of WRKY genes during salt stress revealed delayed induction kinetics for most of the analyzed genes in leaves compared with roots, indicating that plants perceive salt stress and activate their defense machinery rapidly in roots.

**Fig 5:**
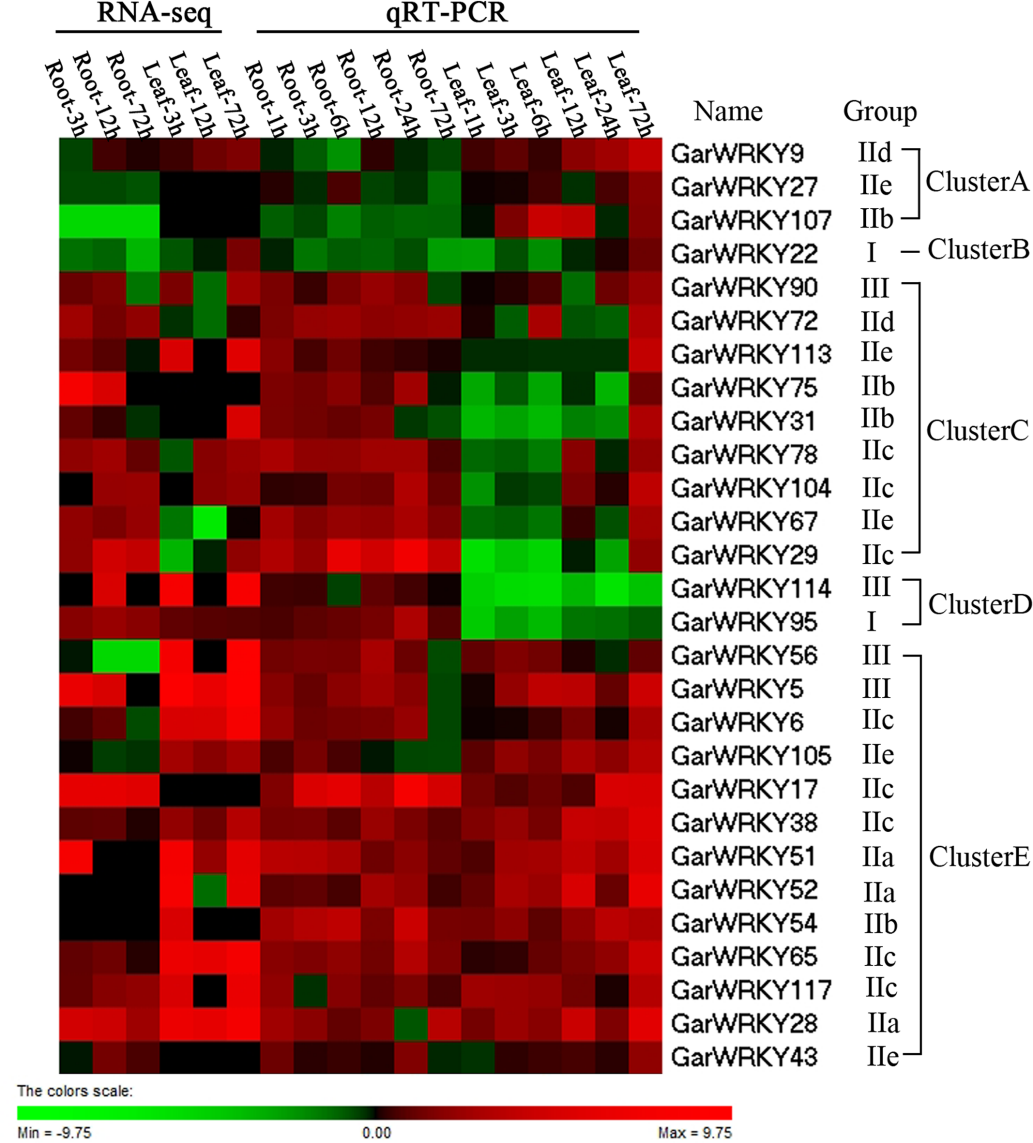
Heat map of the expression patterns of 28 salt-responsive *GarWRKYs* from RNA-Seq and qRT-PCR data. Changes in expression levels are displayed from green (downregulated) to red (upregulated), as shown in the color gradient at the bottom right corner (color figure online). Heat maps were generated and hierarchical clustering was performed using Cluster 3.0 based on log2 fold-change data in response to NaCl stress. The normalized expression values from RNA-Seq data were provided in S4 Table.

Based on hierarchical clustering, the expression patterns of the *GarWRKY* genes were divided into five groups, i.e., Cluster A, Cluster B, Cluster C, Cluster D and Cluster E (Fig 5). Cluster A includes three genes (*GarWRKY9*, *GarWRKY27* and *GarWRKY107*) that were slightly downregulated in roots but upregulated in leaves. Cluster B includes one gene (*GarWRKY*22) that was downregulated in roots and leaves. Cluster C contains nine genes (*GarWRKY90*, *GarWRKY72*, *GarWRKY113*, *GarWRKY75*, *GarWRKY31*, *GarWRKY78*, *GarWRKY104*, *GarWRKY67* and *GarWRKY29*) that were upregulated in roots and downregulated in leaves at early stages (before 24 h) but upregulated at later stage (72 h). Cluster D contains two genes (*GarWRKY95* and *GarWRKY114*) that were upregulated in roots but downregulated in leaves. Finally, Cluster E contains 13 genes (*GarWRKY56*, *GarWRKY5*, *GarWRKY6*, *GarWRKY105*, *GarWRKY17*, *GarWRKY38*, *GarWRKY51*, *GarWRKY52*, *GarWRKY54*, *GarWRKY65*, *GarWRKY117*, *GarWRKY28* and *GarWRKY43*) that were upregulated in roots and leaves.

### Comparison of orthologous *GarWRKY* genes from *Arabidopsis*, rice and soybean

To further implicate the functions of *GarWRKY*s in plant defenses to stresses, the orthologs in *Arabidopsis*, rice and soybean of 28 salt-responsive WRKY members were identified using BLASTP (E < 1e^−20^), and the 58 top hit (17 in *Arabidopsis*, 19 in rice and 22 in soybean, respectively) were collected. A phylogenetic tree was constructed with the MEGA 4.0 software and conserved motif compositions were analyzed by MEME program.

**Fig 6:**
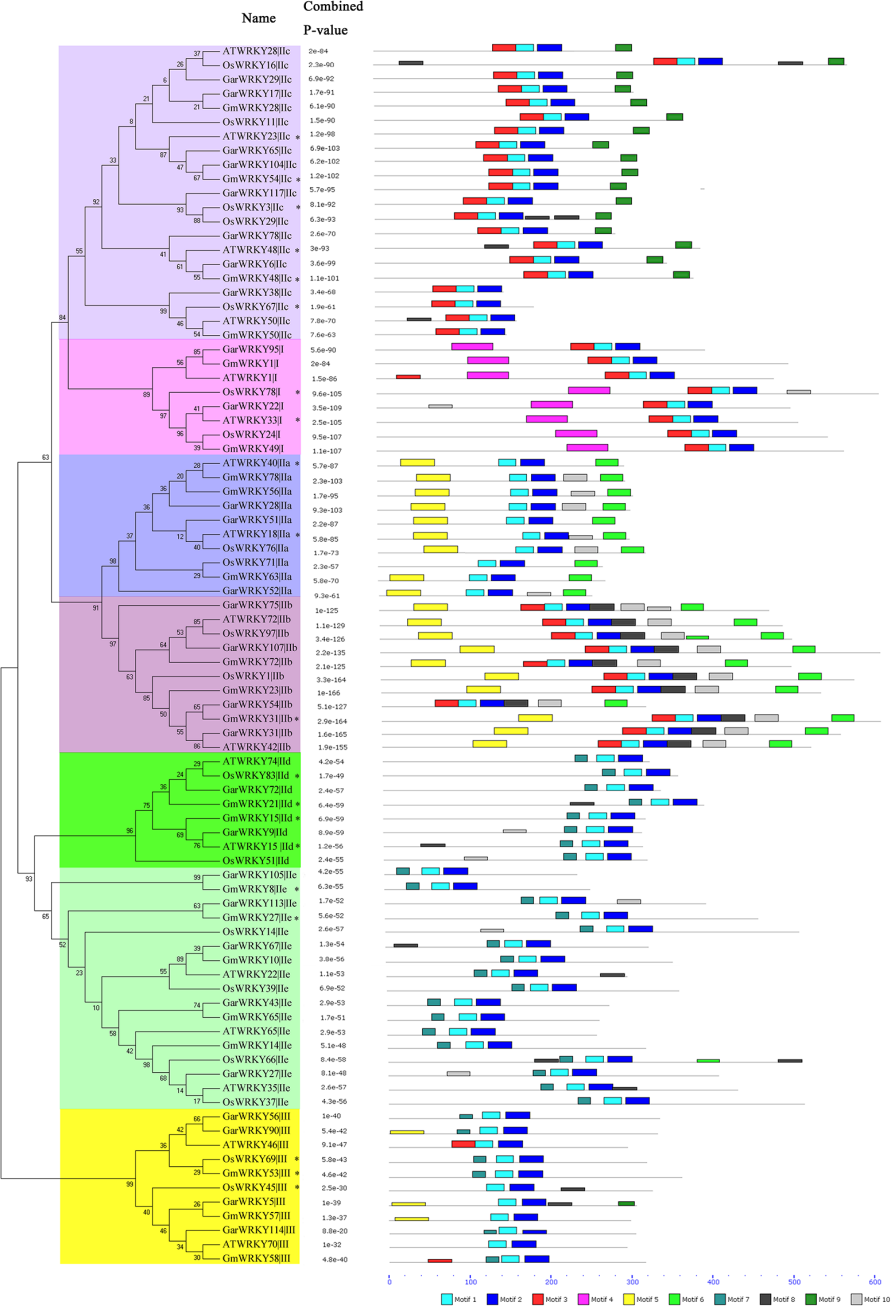
Phylogenetic relationships and motif compositions of 28 salt-responsive GarWRKY members and their orthologs of *Arabidopsis*, rice and soybean. Unrooted phylogenetic tree was constructed by using MEGA4.0. The motifs identified by MEME software are represented by colored boxes. * indicated the genes induced under salt tress.

Our results showed that all 86 WRKY members from different species could be unambiguously classified as Group I, GroupIIa, IIb, IIc, IId, IIe and Group III (Fig 6). In total, 10 motifs, named, motif 1–10, were identified. Motifs 1, 2, 3, 4, were located in the WRKY domains, while the other motifs were dispersed around the WRKY domains. Motifs 1 and 2 were conserved in all WRKY family members; motif 5 was conserved in GroupIIa and IIb except for *GarWRKY54*; motif 4 was specific to Group I WRKY members. In Group I, only *GarWRKY22* and *OsWRKY78* contained motif 10. In Group IId and IIe, motif 10 was found only in *GarWRKY9*, *GarWRKY27*, *GarWRKY113*, *OsWRKY14* and *OsWRKY31*. Overall, similar motif compositions were found in WRKY members of *Arabidopsis*, rice and soybean within the same subgroup, indicating the WRKY members have the similar function in these plants. It is worthwhile to note that 20 orthologous WRKY members in *Arabidopsis*, rice and/or soybean were also induced under salt stress according to previous reports [20–26, 42–44] (Fig 6), further implying these 28 *GarWRKY* members may play an important role in underlying mechanism of gene regulation under salt stress.

### Salt tolerance validation of *GarWRKY17* and *GarWRKY104* in transgenic *Arabidopsis*

**Fig 7:**
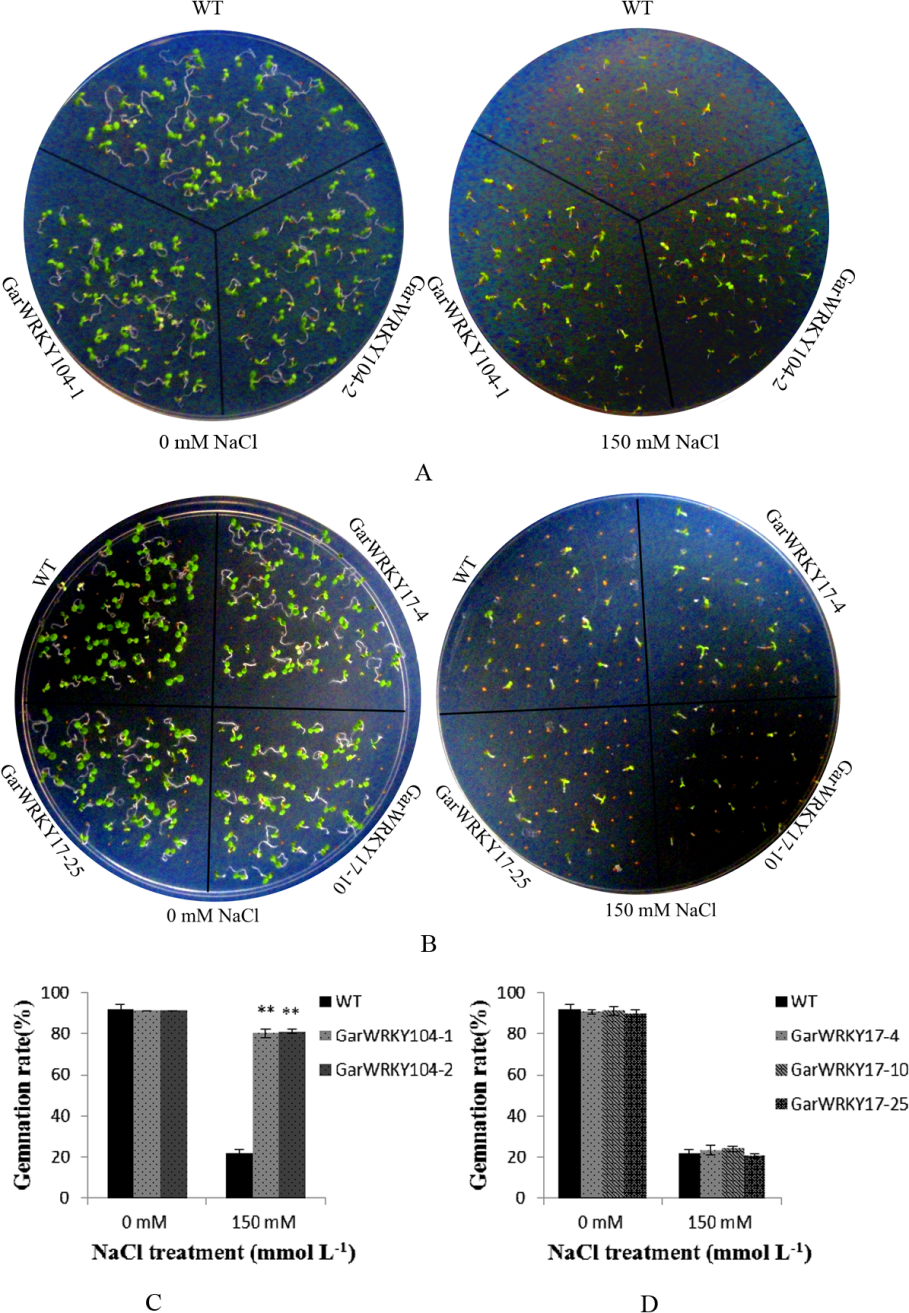
Overexpressing *GarWRKYs Arabidopsis* plants at germination stage under saltstress. (A) Seedlings of the WT and 2 *GarWRKY104*-overexpressing lines germinated on MS medium adding 150 mM NaCl; (B) Seeds from the WT and 3 *GarWRKY17*-overexpressing lines were germinated on MS medium; (C) Germination rate of WT and 2 *GarWRKY104*-overexpressing lines; (D) Germination rate of WT and 3 *GarWRKY17*-overexpressing lines. The data are mean ± SE of three biological replicates. * and ** indicate statistical significance at the 0.05 and 0.01 probability level, respectively.

*GarWRKY17* and *GarWRKY104* showed relatively higher differentially expression level in qRT-PCR analysis (Fig 4). Therefore, they were selected to generate overexpression transgenic *Arabidopsis* plants to further evaluate their function in response to salt stress,. When these lines germinated on the medium adding 150 mM NaCl, the germination rates of two *GarWRKY104*-overexpressing lines (*GarWRKY104-1* and *GarWRKY104-2*) were significantly higher than that of WT (80.0% and 81.3% vs 22.0%), However, no significant difference was observed between WT and three Gar*WRKY17*-overexpressing lines (*GarWRKY17*-4, -10, -25) (Fig 7).

**Fig 8:**
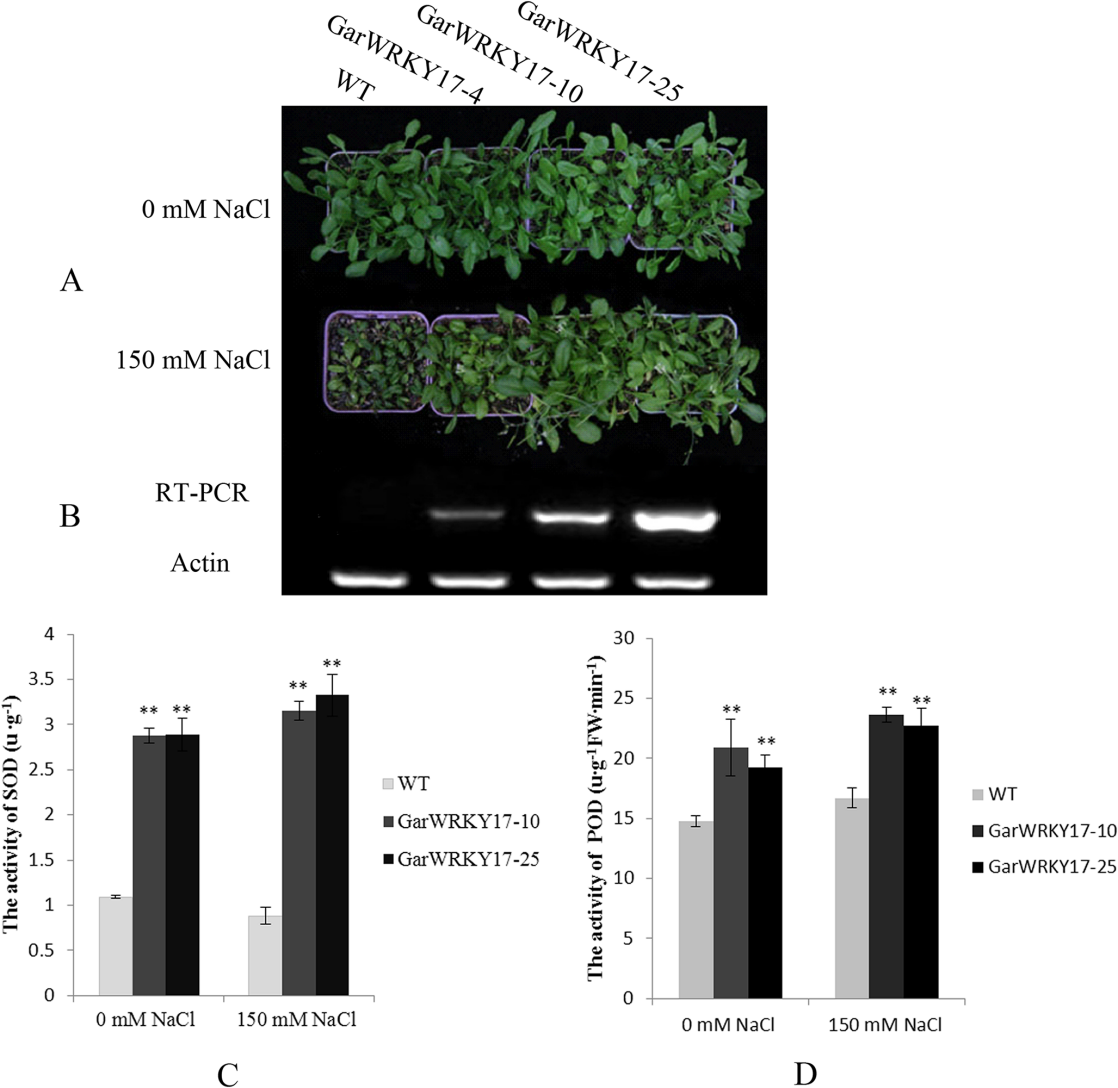
Effect of salt treatment on GarWRKY17-overexpressing Arabidopsis plants at vegetative growth stage. (A) Plants of WT and 3 *GarWRKY17*-overexpressing *Arabidopsis* lines were watered with 150 mM NaCl solution; (B) The expression level of *GarWRKY17* in WT and transgenic T_2_ lines; (C) SOD activity; (D) POD activity. The data are mean ± SE of three biological replicates. * and ** indicate statistical significance at the 0.05 and 0.01 probability level, respectively.

To further validate the salt tolerance of *GarWRKY17* and *GarWRKY104*, 20 day-old overexpressing transgenic plants were treated with150 mM NaCl solution using water as the control. Four weeks later, growth of the WT seedlings was completely inhibited while the *GarWRKY17*–overexpressing seedlings remained green and continued to grow, especially for *GarWRKY17*-10 and *GarWRKY17*-25 lines (Fig 8). Similar to WT, the *GarWRKY104*-overexpressing lines displayed the inhibited growth (data not shown). These data indicate that *GarWRKY17* positively regulate salt tolerance in a wide range of vegetative growth, while *GarWRKY104* exhibited the salt terlance only during seed germination.

To study the physiological response of overexpressing-*GarWRKY17 Arabidopsis* plants to salt stress, two transgenic lines (*GarWRKY17*-10 and *GarWRKY17*-25) which showed better growth than WT plants during vegetative stage under salt stress were selected for further enzyme activities analysis. The activities of SOD and POD were determined in leaves of the control and the salt-treated *Arabidopsis* plants (Fig 8). The activity of SOD was much higher in the transgenic plants as compared to the WT plants with or without salt stress. In particular, the SOD activity in the two transgenic lines increased 2.6 times (*GarWRKY17*-10) and 2.8 times (*GarWRKY17*-25) compared with that in the wild type plants under salt stress. The activity of POD was significant higher in the transgenic plants as compared to the WT plants with or without salt stress. The activity of POD in the two transgenic lines increased 41% (*GarWRKY17*-10) and 36% (*GarWRKY17*-25) compared with that in WT plants under salt stress. These data indicate that overexpression of *GarWRKY17* confer a more efficient antioxidant system to counteract the oxidative stress caused by salt stress.

## Discussion

A few studies have revealed WRKY family members in *G*. *hirsutum* (cultivated species) and *G*. *raimondii* (for which complete genome sequence information is available), and the expression patterns of various WRKY TFs in response to abiotic stress (including salt stress) have been analyzed in upland cotton. As described in detail previously [34], modern cotton cultivars are the result of intensive selection to produce large amounts of specific types of fiber under unstressed conditions. Selection has unintentionally narrowed the genetic variability for salt tolerance. Therefore, improving salt tolerance in upland cotton requires the use of valuable alleles from other *Gossypium* species. In this study, we selected *G*. *aridum*, a D-genome diploid species that grows in the Pacific coastal states of Mexico, to conduct transcriptome-wide identification of WRKY family members. We identified 28 salt-responsive *GarWRKY* genes and validated their expression patterns in *G*. *aridum* using RNA-Seq and qRT-PCR technology. In a recent study, Cai et al. (2014) identified 120 candidate WRKY genes from *G*. *raimondii* and revealed that 17 WRKY genes are significantly induced by salt treatment in *G*. *hirsutum* [31]. Using uniform nomenclature, we found that *WRKY22* in *G*. *hirsutum* and *GarWRKY22* in *G*. *aridum* had similar expression patterns in *G*. *hirsutum* and *G*. *aridum* (significantly downregulated after salt treatment). However, *WRKY6* in *G*. *hirsutum* and *GarWRKY*6 in *G*. *aridum* exhibited opposite expression patterns (downregulated in *G*. *hirsutum* and upregulated in *G*. *aridum*), indicating that some orthologous WRKY genes between *G*. *hirsutum* and *G*. *aridum* may have similar functions in regulating the stress response, while some may not.

In this study, we identified a total of 109 WRKY genes in *G*. *aridum* via de novo transcriptome sequencing of *G*. *aridum* under salt stress. In a recent report, Cai et al. identified 120 candidate WRKY genes from the *G*. *raimondii* genome, while the 11 remaining WRKY genes (*WRKY7*, 16, 33, 42, 44, 49, 89, 96, 102, 109 and 112) were not identified in the current study. Since *G*. *aridum* and *G*. *raimondii* belong to the same subgenome (D genome), they are expected to have the same number of WRKY genes in their genomes. The transcript abundance of these 11 WRKY genes was too low for them to be detected in the RNA-Seq libraries. These genes may be pseudogenes, or they may be expressed at specific developmental stages, under specific conditions or in specific tissues. In Cai’s study, the expression patterns of *WRKY16* and *WRKY44* were examined in different tissues. *WRKY16* was not expressed in roots and was only slightly expressed in leaves, but it was preferentially expressed in fibers. *WRKY44* exhibited very low transcript abundance in roots and leaves but preferential expression in anthers. These WRKY genes, which are expressed under specific conditions or in specific plant tissues, may be involved in the growth and development of different organs.

A number of studies have shown that WRKY genes are induced by salt stress and that overexpression of some WRKY genes alters stress tolerance in plants. In *Arabidopsis*, 18 out of 35 WRKY genes from roots are induced by salt stress, such as *AtWRKY8*, 25, 33, 46, 57 and so on [43]. In rice, 27 WRKY transcription factor genes are induced in response to salt stress, 26 of which are upregulated [44]. In soybean, 22 of 64 WRKY genes are differentially expressed under salt stress [26]. In transgenic rice, overexpression of *OsWRKY11*/01g43650 and *OsWRKY45*/05g2577 results in enhanced salt, heat and drought tolerance [25]. Overexpression of *OsWRKY45* results in enhanced salt and drought tolerance, in addition to increased disease resistance [24]. In *Arabidopsis*, overexpression of either *AtWRKY25* or *AtWRKY33* increases salt tolerance [23], while overexpression of *AtWRKY18* or *AtWRKY60* increases plant sensitivity to salt [45]. *GmWRKY54-*overexpressing plants are more salt and drought tolerant than the control, and *GmWRKY13* overexpression results in increased sensitivity to salt and mannitol stress [26]. In the current study, integrated transcriptome analysis indicated that 28 WRKY genes in *G*. *aridum* were differentially regulated in response to salt stress conditions. To further explore the functions of *GarWRKY*s that may be involved in plant defense responses to salt stress, we identified orthologous pairs among *GarWRKY*s, *AtWRKYs*, *OsWRKYs* and *GmWRKYs* based on full-length protein sequence similarities. Of the 28 salt-induced *GarWRKY* genes, 20 orthologs in *Arabidopsis*, rice and soybean also exhibit significant induction under salt stress. Among these, *GarWRKY22*, *GarWRKY65* and *GarWRKY5* are orthologous to *AtWRKY33*, *GmWRKY54* and *OsWRKY45*, respectively. Motif compositions analysis showed that similar motif compositions were generally shared by WRKY members from *Arabidopsis*, rice and soybean within the same subgroup. These findings suggest that these members are important regulators of the response to salt stress. We also observed there was a possible relation between motif composition and gene function. *GarWRKY22* contained motif 10, which was downregulated under salt stress. However, its orthologous gene in *Arabidopsis* (*AtWRKY33*) didn’t possessed motif 10, which was upregulated under salt stress [23]. Further study is required to clarify whether motif 10 is a key composition in regulating alteration of gene expression and functional divergence in different species.

Recent studies have identified and revealed the expression patterns of various salt-responsive WRKY TFs in cotton [18, 28–34,]. However, functional analysis of these TFs in an important crop such as cotton has rarely been performed. To date, only one WRKY gene in cotton has been functionally characterized [29], while the biological functions of most cotton WRKY genes remain largely unknown. In this study, we performed the first transcriptome-wide analysis of salt-responsive members of the WRKY family in *G*. *aridum*, a highly salt-tolerant wild species. Functional analysis by overexpression of *GarWRKY17* and *GarWRKY104* in *Arabidopsis* indicated they could positively regulate salt tolerance in *Arabidopsis* different development stages.This study demonstrates that a number of WRKY genes might be involved in the response to salt stress, and it provides clues for the selection of candidate genes for use in improving the stress tolerance of upland cotton.

## Supporting Information

S1 Table Characterization of 109 *GarWRKY* members. (DOC)

S2 Table Primers used for *GarWRKY* gene cloning. (DOC)

S3 Table Primers used for qRT-PCR. (DOC)

S4 Table The normalized expression values of 28 salt-responsive *GarWRKYs* from RNA-Seq. (DOC)
