# Supplementary material for: Transcriptome-Wide Identification of Salt-Responsive Members of the WRKY Gene Family in Gossypium aridum
Source: PLoS One. 2015 May 7;10(5):e0126148. doi: 10.1371/journal.pone.0126148 (PMC4423833; doi:10.1371/journal.pone.0126148)
Supplement: S1 Table — (DOC) [file pone.0126148.s001.doc]

**Table S1 Characterization of 109 *GarWRKY* members**

| Gene symbol | | chromosome | WRKY domain | | Group |
| --- | --- | --- | --- | --- | --- |
| G.ardium | G.raimondii |  | Conserved heptapeptide | Zinc-ﬁnger type |  |
| GarWRKY001 | WRKY001 | 2 | WRKYGQK | C-X_5_-CX_23_-HXH | Ⅱb |
| GarWRKY002 | WRKY002 | 2 | WRKYGQK/WRKYGQK | C-X_4_-CX_23_-HXH/C-X_4_-CX_22_-HXH | Ⅰ |
| GarWRKY003 | WRKY003 | 2 | WRKYGQK | C-X_4_-CX_23_-HXH | Ⅱc |
| GarWRKY004 | WRKY004 | 2 | WRKYGQK | C-X_5_-CX_23_-HXH | Ⅱb |
| GarWRKY005 | WRKY005 | 5 | WRKYGQK | C-X_7_-CX_23_-HXC | Ⅲ |
| GarWRKY006 | WRKY006 | 5 | **WRKYGHK** | C-X_4_-CX_23_-HXH | Ⅱc |
| GarWRKY008 | WRKY008 | 5 | WRKYGQK | C-X_4_-CX_23_-HXH | Ⅱc |
| GarWRKY009 | WRKY009 | 3 | WRKYGQK | C-X_5_-CX_23_-HXH | Ⅱd |
| GarWRKY010 | WRKY010 | 3 | **WRKYGKK** | C-X_4_-CX_23_-HXH | Ⅱc |
| GarWRKY011 | WRKY011 | 3 | WRKYGQK | C-X_4_-CX_23_-HXH | Ⅱc |
| GarWRKY012 | WRKY012 | 3 | WRKYGQK | C-X_5_-CX_23_-HXH | Ⅱe |
| GarWRKY013 | WRKY013 | 3 | WRKYGQK/WRKYGQK | C-X_4_-CX_23_-HXH/C-X_4_-CX_22_-HXH | Ⅰ |
| GarWRKY014 | WRKY014 | 12 | WRKYGQK | C-X_7_-CX_23_-HXC | Ⅲ |
| GarWRKY015 | WRKY015 | 12 | WRKYGQK | C-X_7_-CX_23_-HXC | Ⅲ |
| GarWRKY017 | WRKY017 | 12 | WRKYGQK | C-X_4_-CX_23_-HXH | Ⅱc |
| GarWRKY018 | WRKY018 | 12 | WRKYGQK/WRKYGQK | C-X_4_-CX_23_-HXH/C-X_4_-CX_22_-HXH | Ⅰ |
| GarWRKY019 | WRKY019 | 12 | WRKYGQK | C-X_5_-CX_23_-HXH | Ⅱb |
| GarWRKY020 | WRKY020 | 12 | WRKYGQK | C-X4-CX23-HXH | Ⅱc |
| GarWRKY021 | WRKY021 | 12 | WRKYGQK | C-X4-CX23-HXH | Ⅱc |
| GarWRKY022 | WRKY022 | 12 | WRKYGQK/WRKYGQK | C-X4-CX23-HXH/C-X4-CX22-HXH | Ⅰ |
| GarWRKY023 | WRKY023 | 12 | WRKYGQK/WRKYGQK | C-X4-CX23-HXH/C-X4-CX22-HXH | Ⅰ |
| GarWRKY024 | WRKY024 | 9 | WRKYGQK | C-X_5_-CX_23_-HXH | Ⅱa |
| GarWRKY025 | WRKY025 | 9 | WRKYGQK | C-X_5_-CX_23_-HXH | Ⅱa |
| GarWRKY026 | WRKY026 | 9 | WRKYGQK | C-X_5_-CX_23_-HXH | Ⅱd |
| GarWRKY027 | WRKY027 | 9 | WRKYGQK | C-X_5_-CX_23_-HXH | Ⅱe |
| GarWRKY028 | WRKY028 | 9 | WRKYGQK | C-X_5_-CX_23_-HXH | Ⅱa |
| GarWRKY029 | WRKY029 | 9 | WRKYGQK | C-X_4_-CX_23_-HXH | Ⅱc |
| GarWRKY030 | WRKY030 | 9 | WRKYGQK | C-X_5_-CX_23_-HXH | Ⅱe |
| GarWRKY031 | WRKY031 | 9 | WRKYGQK | C-X_5_-CX_2_3-HXH | Ⅱb |
| GarWRKY032 | WRKY032 | 9 | WRKYGQK | C-X_5_-CX_23_-HXH | Ⅱd |
| GarWRKY034 | WRKY034 | 9 | WRKYGQK | C-X_5_-CX_23_-HXH | Ⅱd |
| GarWRKY035 | WRKY035 | 9 | WRKYGQK | **C-X_7_-CX_24_-HXC** | Ⅲ |
| GarWRKY036 | WRKY036 | 9 | WRKYGQK/WRKYGQK | C-X_4_-CX_23_-HXH/C-X_4_-CX_22_-HXH | Ⅰ |
| GarWRKY037 | WRKY037 | 10 | WRKYGQK | C-X_5_-CX_23_-HXH | Ⅱd |
| GarWRKY038 | WRKY038 | 10 | **WRKYGKK** | C-X_4_-CX_23_-HXH | Ⅱc |
| GarWRKY039 | WRKY039 | 10 | WRKYGQK | C-X_4_-CX_23_-HXH/C-X_4_-CX_22_-HXH | Ⅰ |
| GarWRKY040 | WRKY040 | 10 | WRKYGQK | C-X_5_-CX_23_-HXH | Ⅱa |
| GarWRKY041 | WRKY041 | 10 | WRKYGQK | C-X_5_-CX_23_-HXH | Ⅱd |
| GarWRKY043 | WRKY043 | 10 | WRKYGQK | C-X_5_-CX_23_-HXH | Ⅱe |
| GarWRKY045 | WRKY045 | 10 | WRKYGQK | C-X_5_-CX_23_-HXH | Ⅱb |
| GarWRKY046 | WRKY046 | 10 | WRKYGQK | C-X_7_-CX_23_-HXC | Ⅲ |
| GarWRKY047 | WRKY047 | 10 | WRKYGQK | C-X_5_-CX_23_-HXH | Ⅱa |
| GarWRKY048 | WRKY048 | 1 | WRKYGQK | C-X_4_-CX_23_-HXH | Ⅱd |
| GarWRKY050 | WRKY050 | 1 | WRKYGQK/WRKYGQK | C-X_4_-CX_23_-HXH/C-X_4_-CX_22_-HXH | Ⅰ |
| GarWRKY051 | WRKY051 | 1 | WRKYGQK | C-X_5_-CX_23_-HXH | Ⅱa |
| GarWRKY052 | WRKY052 | 1 | WRKYGQK | C-X_5_-CX_23_-HXH | Ⅱa |
| GarWRKY053 | WRKY053 | 1 | WRKYGQK | C-X_4_-CX_23_-HXH | Ⅱc |
| GarWRKY054 | WRKY054 | 1 | WRKYGQK | C-X_5_-CX_23_-HXH | Ⅱb |
| GarWRKY055 | WRKY055 | 1 | WRKYGQK | C-X_5_-CX_23_-HXH | Ⅱb |
| GarWRKY056 | WRKY056 | 1 | WRKYGQK | C-X_7_-CX_23_-HXC | Ⅲ |
| GarWRKY057 | WRKY057 | 1 | WRKYGQK/WRKYGQK | C-X_4_-CX_23_-HXH/C-X_4_-CX_22_-HXH | Ⅰ |
| GarWRKY058 | WRKY058 | 1 | WRKYGQK | C-X_5_-CX_23_-HXH | Ⅱb |
| GarWRKY059 | WRKY059 | 1 | WRKYGQK/WRKYGQK | C-X_4_-CX_23_-HXH/C-X_4_-CX_22_-HXH | Ⅰ |
| GarWRKY060 | WRKY060 | 1 | WRKYGQK | C-X_4_-CX_23_-HXH | Ⅱc |
| GarWRKY061 | WRKY061 | 4 | WRKYGQK/WRKYGQK | C-X_4_-CX_23_-HXH/C-X_4_-CX_22_-HXH | Ⅰ |
| GarWRKY062 | WRKY062 | 4 | WRKYGQK/WRKYGQK | C-X_4_-CX_23_-HXH/C-X_4_-CX_22_-HXH | Ⅰ |
| GarWRKY063 | WRKY063 | 4 | WRKYGQK | C-X_4_-CX_23_-HXH | Ⅱc |
| GarWRKY064 | WRKY064 | 4 | WRKYGQK | C-X_7_-CX_23_-HXC | Ⅲ |
| GarWRKY065 | WRKY065 | 4 | WRKYGQK | C-X_4_-CX_23_-HXH | Ⅱc |
| GarWRKY066 | WRKY066 | 4 | WRKYGQK | C-X_4_-CX_23_-HXH | Ⅱc |
| GarWRKY067 | WRKY067 | 4 | WRKYGQK | C-X_4_-CX_23_-HXH | Ⅱe |
| GarWRKY068 | WRKY068 | 4 | WRKYGQK | C-X_4_-CX_23_-HXH | Ⅱc |
| GarWRKY069 | WRKY069 | 4 | WRKYGQK | C-X_5_-CX_23_-HXH | Ⅱd |
| GarWRKY070 | WRKY070 | 4 | WRKYGQK | C-X_5_-CX_23_-HXH | Ⅱd |
| GarWRKY071 | WRKY071 | 4 | WRKYGQK | C-X_5_-CX_23_-HXH | Ⅱd |
| GarWRKY072 | WRKY072 | 5 | WRKYGQK | C-X_5_-CX_23_-HXH | Ⅱd |
| GarWRKY073 | WRKY073 | 6 | WRKYGQK | C-X_4_-CX_23_-HXH | Ⅱc |
| GarWRKY074 | WRKY074 | 6 | WRKYGQK/WRKYGQK | C-X_4_-CX_23_-HXH/C-X_4_-CX_22_-HXH | Ⅰ |
| GarWRKY075 | WRKY075 | 6 | WRKYGQK | C-X_5_-CX_23_-HXH | Ⅱb |
| GarWRKY076 | WRKY076 | 6 | WRKYGQK | C-X_4_-CX_23_-HXH | Ⅱc |
| GarWRKY077 | WRKY077 | 6 | WRKYGQK | C-X_4_-CX_23_-HXH | Ⅱc |
| GarWRKY078 | WRKY078 | 6 | WRKYGQK | C-X_4_-CX_23_-HXH | Ⅱc |
| GarWRKY079 | WRKY079 | 11 | WRKYGQK | C-X_5_-CX_23_-HXH | Ⅱe |
| GarWRKY080 | WRKY080 | 11 | WRKYGQK | C-X_5_-CX_23_-HXH | Ⅱb |
| GarWRKY081 | WRKY081 | 11 | WRKYGQK | C-X_5_-CX_23_-HXH | Ⅱe |
| GarWRKY082 | WRKY082 | 11 | WRKYGQK | C-X_4_-CX_23_-HXH | Ⅱc |
| GarWRKY083 | WRKY083 | 11 | WRKYGQK | C-X_4_-CX_23_-HXH | Ⅱc |
| GarWRKY084 | WRKY084 | 11 | **WRKYGKK** | C-X_4_-CX_23_-HXH | Ⅱc |
| GarWRKY085 | WRKY085 | 11 | WRKYGQK | C-X_4_-CX_23_-HXH | Ⅱc |
| GarWRKY086 | WRKY86 | 7 | WRKYGQK | C-X_5_-CX_23_-HXH | Ⅱe |
| GarWRKY087 | WRKY87 | 7 | WRKYGQK | C-X_7_-CX_23_-HXC | Ⅲ |
| GarWRKY88 | WRKY88 | 7 | WRKYGQK | C-X_5_-CX_23_-HXH | Ⅱe |
| GarWRKY90 | WRKY90 | 7 | WRKYGQK | C-X_7_-CX_23_-HXC | Ⅲ |
| GarWRKY91 | WRKY91 | 7 | WRKYGQK | C-X_5_-CX_23_-HXH | Ⅱb |
| GarWRKY92 | WRKY92 | 7 | WRKYGQK | C-X_4_-CX_23_-HXH | Ⅱc |
| GarWRKY93 | WRKY93 | 7 | WRKYGQK | C-X_5_-CX_23_-HXH | Ⅱe |
| GarWRKY94 | WRKY94 | 7 | WRKYGQK | C-X_5_-CX_23_-HXH | Ⅱd |
| GarWRKY95 | WRKY95 | 7 | WRKYGQK/ WRKYGQK | C-X_4_-CX_23_-HXH/C-X_4_-CX_22_-HXH | Ⅰ |
| GarWRKY97 | WRKY97 | 7 | WRKYGQK | C-X_5_-CX_23_-HXH | Ⅱd |
| GarWRKY98 | WRKY98 | 7 | **WRKYGKK** | C-X_4_-CX_23_-HXH | Ⅱc |
| GarWRKY99 | WRKY99 | 7 | WRKYGQK | C-X_4_-CX_23_-HXH | Ⅱc |
| GarWRKY100 | WRKY100 | 7 | WRKYGQK | C-X_5_-CX_23_-HXH | Ⅱd |
| GarWRKY101 | WRKY101 | 8 | WRKYGQK | C-X_5_-CX_23_-HXH | Ⅱb |
| GarWRKY103 | WRKY103 | 8 | WRKYGQK | C-X_5_-CX_23_-HXH | Ⅱb |
| GarWRKY104 | WRKY104 | 8 | WRKYGQK | C-X_4_-CX_23_-HXH | Ⅱc |
| GarWRKY105 | WRKY105 | 8 | WRKYGQK | C-X_5_-CX_23_-HXH | Ⅱe |
| GarWRKY106 | WRKY106 | 8 | WRKYGQK | C-X_4_-CX_23_-HXH | Ⅱc |
| GarWRKY107 | WRKY107 | 8 | WRKYGQK | C-X_5_-CX_23_-HXH | Ⅱb |
| GarWRKY108 | WRKY108 | 8 | WRKYGQK | C-X_7_-CX_23_-HXC | Ⅲ |
| GarWRKY110 | WRKY110 | 8 | WRKYGQK | C-X_5_-CX_23_-HXH | Ⅱe |
| GarWRKY111 | WRKY111 | 8 | WRKYGQK | C-X_7_-CX_23_-HXC | Ⅲ |
| GarWRKY113 | WRKY113 | 8 | WRKYGQK | C-X_5_-CX_23_-HXH | Ⅱe |
| GarWRKY114 | WRKY114 | 13 | WRKYGQK | **C-X_7_-CX_24_-HXC** | Ⅲ |
| GarWRKY115 | WRKY115 | 13 | WRKYGQK | C-X_5_-CX_23_-HXH | Ⅱb |
| GarWRKY116 | WRKY116 | 13 | WRKYGQK/WRKYGQK | C-X_4_-CX_23_-HXH/C-X_4_-CX_22_-HXH | Ⅰ |
| GarWRKY117 | WRKY117 | 13 | WRKYGQK | C-X_4_-CX_22_-HXH | Ⅱc |
| GarWRKY118 | WRKY118 | 13 | WRKYGQK | C-X_5_-CX_23_-HXH | Ⅱd |
| GarWRKY119 | WRKY119 | 13 | WRKYGQK/WRKYGQK | C-X_4_-CX_23_-HXH/C-X_4_-CX_22_-HXH | Ⅰ |
| GarWRKY120 | WRKY120 | 13 | WRKYGQK/WRKYGQK | C-X_4_-CX_23_-HXH/C-X_4_-CX_22_-HXH | Ⅰ |
