# Supplementary material for: Transcriptome-Wide Identification of Salt-Responsive Members of the WRKY Gene Family in Gossypium aridum
Source: PLoS One. 2015 May 7;10(5):e0126148. doi: 10.1371/journal.pone.0126148 (PMC4423833; doi:10.1371/journal.pone.0126148)
Supplement: S2 Table — (DOC) [file pone.0126148.s002.doc]

**Table S2. Primers used for *GarWRKY* genes cloning**

| Primers name | Sequences(5’-3’) |
| --- | --- |
| GarWRKY5F | AGCAAACATGTCTTGGAAC |
| GarWRKY5R | CGGCCTTTCAAAACTGA |
| GarWRKY6F | ATGGAGAAGAAGAACGAGAA |
| GarWRKY6R | CCTACTGATCTTCTTCCTTTGC |
| GarWRKY9F | ATGGTTTTATTTGCAGACATGGC |
| GarWRKY9R | CTATGAAGATTCAAGTATGGTGG |
| GarWRKY17F | ATGTCTTCAAATGAAAAGAACGATCC |
| GarWRKY17R | TCATTCATATGGCGCCTGACTACG |
| GarWRKY22F | ATGGCTGCTTCATCATCATCTGC |
| GarWRKY22R | TCAAGACAGTAATCCGTCCA |
| GarWRKY27F | TGGATAACTATCAAGGTGAAGGTGA |
| GarWRKY27F | TCAGAAAGAAGCATCGTTGT |
| GarWRKY28F | AGCTATGGATACTTCACTGGAT |
| GarWRKY28R | TTATTCAACTGGACTTTGCTG |
| GarWRKY29F | CGATCTCTCCATGTCGGAT |
| GarWRKY29R | GGAGACTCATGGTTCATGTTTA |
| GarWRKY31F | ATTTATGGAGAAGGGATGGG |
| GarWRKY31R | GGCATTACTTGTTGTCGTTG |
| GarWRKY38F | ATGTCTAGCAGCAATTTTAATCCGC |
| GarWRKY38R | ATTCAAGAAACGCTACGGT |
| GarWRKY43F | GCAGATCCCAACTTGTGGTCC |
| GarWRKY43R | ATGGACGCTGGTGACAGTAAT |
| GarWRKY51F | AAGTGGGATATGCCTCTCA |
| GarWRKY51R | CATCTCATCTATATAACTCTCCCTG |
| GarWRKY52F | ATGGATGTTCCCAGCTCTGT |
| GarWRKY52R | TTAGTTTGTTGGTGGTTTAGGG |
| GarWRKY54F | ATGGCGAAAACCAGTGGACT |
| GarWRKY54R | CTACATTGCATTGCTAGTGTTAGTG |
| GarWRKY56F | ATGGAAAAAACGATGGGT |
| GarWRKY56R | TTACTGTAAAAAATTAGTGAAGAAC |
| GarWRKY65F | TTCATGGAGAGTAAAGAGGGT |
| GarWRKY65R | CTACTCTTCTTTCAGCATATGG |
| GarWRKY67F | ATGGATGTGGATTGGGATT |
| GarWRKY67R | CGAGTTTTAGATGCCACCG |
| GarWRKY72F | ATGGACATGGAGGGGGTTGA |
| GarWRKY72R | TCATGTATGGGCAGATTGTGTAGAG |
| GarWRKY75F | GTTGATGAATATGGCAGATG |
| GarWRKY75R | CTATCCATTAATTCTTTCCTTG |
| GarWRKY78R | ATGAGATTTTCCGATGAG |
| GarWRKY78F | CACTTGTTCATTAGCCTCGTTC |
| GarWRKY90R | TTAATGGAGCAAGAGGCTC |
| GarWRKY90F | GATCAATAGAAGAACTCAGGGT |
| GarWRKY95R | ATAGATGGTTCCGTCAGGG |
| GarWRKY95F | CCACTTCAAGGACCTGATATAG |
| GarWRKY104F | CCTATATGCATACATTCATGGAG |
| GarWRKY104R | CTACTCTTCTTTCAGCATATGAG |
| GarWRKY105F | TTGCAATGGACGGTTCTAT |
| GarWRKY105R | ATCAAGGAGGGACAACAGG |
| GarWRKY107F | ATGGAGGAGAATTTGAAGAAGAG |
| GarWRKY107R | TCAATTGGTGTGATTCCCAG |
| GarWRKY113F | ATGGCTGAGGATTGGGAT |
| GarWRKY113R | ATGGCTGAGGATTGGGAT |
| GarWRKY114F | ATGGGTACACTTTCAGCTTG |
| GarWRKY114R | GTTACAACACACACTCATCACTTC |
| GarWRKY117F | ATGATGGAAGATAAAGAGGGG |
| GarWRKY117R | TCACGTCTTACGCATGCCA |
