# Supplementary material for: Transcriptome-Wide Identification of Salt-Responsive Members of the WRKY Gene Family in Gossypium aridum
Source: PLoS One. 2015 May 7;10(5):e0126148. doi: 10.1371/journal.pone.0126148 (PMC4423833; doi:10.1371/journal.pone.0126148)
Supplement: S3 Table — (DOC) [file pone.0126148.s003.doc]

**Table S3. Primers used for RT-PCR and qRT-PCR**

| Primers name | Sequences(5’-3’) |
| --- | --- |
| GarWRKY5-RT-F | GCCTTGTCATTTCATGGTGGA |
| GarWRKY5-RT-R | GGGTTGTCGTTGCCTTGC |
| GarWRKY6-RT-F | GGCAGTGGTGGAGATAAGTG |
| GarWRKY6-RT-R | CTGGTAATGGAGGTGAGGA |
| GarWRKY9-RT-F | GTTCCAATCTTCTCCGACG |
| GarWRKY9-RT-R | CGACCCAAAAGAGAGATGAC |
| GarWRKY17-RT-F | CTTCCCATTCTTTGACGACAACAGC |
| GarWRKY17-RT-R | TGTCAGTAGCCGTATTGTCATCCAT |
| GarWRKY22-RT-F | ACTTGTAATAACTGCGTGG |
| GarWRKY22-RT-R | CCAATACCATCAAACATCACAG |
| GarWRKY27-RT-F | CATCATTGGCGAATCACAG |
| GarWRKY27-RT-F | AATGGTTGGGCAAGGTAAG |
| GarWRKY28-RT-F | GGCAAAACCAAGCCCAGTG |
| GarWRKY28-RT-R | GAAGTAGCCATCTGTTCCACCAA |
| GarWRKY29-RT-F | GTTACGGCGACTCTTAATTCTTCTG |
| GarWRKY29-RT-R | GCTGCCCATCTTTCTTACTCTTG |
| GarWRKY31-RT-F | TCTGATGGCTGGGGTCCTAA |
| GarWRKY31-RT-R | TTCCTCATGGTGGCCTCTGTA |
| GarWRKY38-RT-F | GAGTTCCCCGAGGACTGGACTTTTG |
| GarWRKY38-RT-R | TTTCAGATACTTGGAGGAGGCTG |
| GarWRKY43-RT-F | GAAACTAACGGTTCCTCCTC |
| GarWRK43-RT-R | TGGTATGGTATTCCCGATG |
| GarWRKY51-RT-F | TCAACATCAATCCTTTCCATCTCC |
| GarWRKY51-RT-R | CAAATCGTTGTAGCAGTGACCCA |
| GarWRKY52-RT-F | AGCTCCGCCACGGCATC |
| GarWRKY52-RT-R | CCGCTGCTGTTTTCGCTTG |
| GarWRKY54-RT-F | GCTCCTCAAGTATGGCAACTATTTC |
| GarWRKY54-RT-R | AACTGCAAGCCTGAGAATTTGGACT |
| GarWRKY56-RT-F | AACTTGTCACCGTTGAGAGG |
| GarWRKY56-RT-R | GGATGAAGATGGATGGAGG |
| GarWRKY65-RT-F | GGGTTTATGGAGTTGTTGGGTG |
| GarWRKY65-RT-R | GAAGCTGATGAAATCGAGGACG |
| GarWRKY67-RT-F | GCCAGAAGCCTTTCACTCCA |
| GarWRKY67-RT-R | AACCACAACCCCTTCATCCAC |
| GarWRKY72-RT-F | CAAGAAGGTGTTCATTTAGGGGAG |
| GarWRKY72-RT-R | CTAATTGCAGGCACTTTTATTGTCC |
| GarWRKY75-RT-F | CAAGGCAATCACATCAGACCC |
| GarWRKY75-RT-R | GCCGCCTGCATCAAATTC |
| GarWRKY78-RT-F | CCTACACTTGTTCATTAGCCTC |
| GarWRKY78-RT-R | ATCGGTTTGACCCCATC |
| GarWRKY90-RT-F | CCTCCCTATTGTCCAAGTCCTCTG |
| GarWRKY90-RT-R | CTCCGAAAAGACAGCACCAGC |
| GarWRKY95-RT-F | TGTCGGCAACATTCTGTATCCTA |
| GarWRKY95-RT-R | TCACCAAATTGAGCCAAGAAGTG |
| GarWRKY104-RT-F | TGCTACACCTAACTCTTCCTCG |
| GarWRKY104-RT-R | TGTCCTCTTCACTTTCAACTG |
| GarWRKY105-RT-F | AACTCAGGCATCCAAAAGAAGG |
| GarWRKY105-RT-R | CATGAATCATAAGGTGGTCCGTC |
| GarWRKY107-RT-F | TAACTTCCACCGCCAACC |
| GarWRKY107-RT-R | GAGGATGAAGATGGTGTTGA |
| GarWRKY113-RT-F | ACCTCCACTGTTTCTTGCTCCTC |
| GarWRKY113-RT-R | TTTCTTCTTCACCTCCATTATCACC |
| GarWRKY114-RT-F | ACATCTGTTAATGTTGCTTCTTCCC |
| GarWRKY114-RT-R | TGCTTGTTCTGCCCTCTTTCTC |
| GarWRKY117-RT-F | TTAGGAAACTACATACCAGCAACCA |
| GarWRKY117-RT-R | ACTCATCGCCTTCTTTGAACATTAC |
| Actin-RT-F: | ATCCTCCGTCTTGACCTTG |
| Actin-RT-R: | TGTCCGTCAGGCAACTCAT |
