# Supplementary material for: Transcriptome-Wide Identification of Salt-Responsive Members of the WRKY Gene Family in Gossypium aridum
Source: PLoS One. 2015 May 7;10(5):e0126148. doi: 10.1371/journal.pone.0126148 (PMC4423833; doi:10.1371/journal.pone.0126148)
Supplement: S4 Table — (DOC) [file pone.0126148.s004.doc]

| Gene | EST | TPM-leaf0 | TPM-leaf3 | TPM-leaf12 | TPM-leaf72 | TPM-root0 | TPM-root3 | TPM-root12 | TPM-root72 |
| --- | --- | --- | --- | --- | --- | --- | --- | --- | --- |
| GarWRKY5 | Unigene91622_G3 | 0.01 | 4.27 | 0.87 | 8.63 | 0.01 | 0.84 | 0.35 | 0.01 |
| GarWRKY6 | Unigene18670_G3 | 0.01 | 0.34 | 0.35 | 2.29 | 5.16 | 7.18 | 10.28 | 3.38 |
| GarWRKY9 | Unigene69321_G3 | 0.52 | 0.68 | 1.22 | 1.58 | 1.38 | 1.00 | 1.92 | 1.52 |
| GarWRKY17 | Unigene38495_G3 | 0.01 | 0.01 | 0.01 | 0.01 | 0.01 | 0.67 | 0.70 | 0.51 |
| GarWRKY22 | Unigene79373_G3 | 2.74 | 1.62 | 2.58 | 7.31 | 2.13 | 0.87 | 1.06 | 0.21 |
| GarWRKY27 | Unigene36404_G3 | 0.01 | 0.01 | 0.01 | 0.01 | 6.37 | 4.34 | 4.36 | 3.89 |
| GarWRKY28 | Unigene85051_G3 | 0.01 | 1.03 | 0.69 | 1.76 | 0.34 | 9.02 | 7.32 | 1.86 |
| GarWRKY29 | Unigene13748_G3 | 0.01 | 0.04 | 0.25 | 0.15 | 0.01 | 0.01 | 0.09 | 0.4 |
| GarWRKY31 | Unigene80304_G3 | 0.01 | 0.01 | 0.01 | 0.35 | 3.61 | 7.02 | 4.53 | 3.05 |
| GarWRKY38 | Unigene40630_G3 | 1.55 | 6.83 | 3.47 | 15.32 | 4.13 | 7.52 | 8.01 | 4.57 |
| GarWRKY43 | Unigene65888_G3 | 0.01 | 0.01 | 0.01 | 0.01 | 0.34 | 0.33 | 0.87 | 0.51 |
| GarWRKY51 | Unigene93932_G3 | 0.86 | 152.23 | 3.82 | 53.89 | 0.01 | 2.01 | 0.01 | 0.01 |
| GarWRKY52 | Unigene93445_G3 | 0.01 | 2.39 | 0.01 | 0.7 | 0.01 | 0.01 | 0.01 | 0.01 |
| GarWRKY54 | Unigene12107_G3 | 0.01 | 0.34 | 0.01 | 0.01 | 0.01 | 0.01 | 0.01 | 0.01 |
| GarWRKY56 | Unigene18520_G3 | 0.01 | 1.88 | 0.01 | 0.01 | 0.01 | 0.33 | 0.01 | 0.01 |
| GarWRKY65 | Unigene87872_G3 | 0.01 | 1.03 | 0.69 | 1.94 | 10.15 | 19.55 | 24.21 | 11.34 |
| GarWRKY67 | Unigene54335_G3 | 0.86 | 0.34 | 0.01 | 0.01 | 0.01 | 9.19 | 6.62 | 10.49 |
| GarWRKY72 | Unigene23081_G3 | 28.25 | 23.75 | 12.51 | 33.99 | 1.03 | 5.85 | 2.61 | 4.23 |
| GarWRKY75 | Unigene94879_G3 | 0.01 | 0.01 | 0.01 | 0.01 | 0.01 | 2.01 | 0.35 | 0.01 |
| GarWRKY78 | Unigene93063_G3 | 0.2 | 0.12 | 0.68 | 1.03 | 0.01 | 0.04 | 0.06 | 0.02 |
| GarWRKY90 | Unigene2576_G3 | 4.65 | 12.81 | 2.08 | 26.77 | 1.72 | 3.68 | 4.88 | 0.68 |
| GarWRKY95 | Unigene34185_G3 | 1.72 | 3.25 | 2.95 | 2.82 | 1.89 | 6.52 | 9.23 | 6.6 |
| GarWRKY104 | Unigene76597_G3 | 0.01 | 0.01 | 0.04 | 0.05 | 0.01 | 0.01 | 0.05 | 0.05 |
| GarWRKY105 | Unigene18122_G3 | 6.72 | 42.71 | 24.5 | 41.56 | 9.12 | 9.19 | 6.97 | 7.45 |
| GarWRKY107 | Unigene47099_G3 | 0.01 | 0.01 | 0.01 | 0.01 | 1.55 | 2.51 | 3.48 | 1.02 |
| GarWRKY113 | Unigene95644_G3 | 0.01 | 0.01 | 0.01 | 0.53 | 6.19 | 15.87 | 10.8 | 5.92 |
| GarWRKY114 | Unigene19806_G3 | 0.01 | 2.39 | 0.01 | 2.99 | 0.01 | 0.01 | 0.01 | 0.01 |
| GarWRKY117 | Unigene31056_G3 | 0.01 | 0.34 | 0.01 | 0.88 | 0.86 | 1.67 | 2.96 | 3.55 |

**TableS4. The normalized expression values of 28 salt-responsive *GarWRKYs***

**from RNA-Seq**
